# Postpartum symptoms of anxiety, depression and stress: differential relationships to women’s cortisol profiles

**DOI:** 10.1007/s00737-024-01421-9

**Published:** 2024-01-12

**Authors:** Sandra J. Weiss, Ling Xu

**Affiliations:** Department of Community Health Systems, University of California, San Francisco, CA USA

**Keywords:** Anxiety, Depression, Stress, Postpartum, Cortisol

## Abstract

**Purpose:**

Women are at high risk of stress, anxiety, and depression during the postpartum but the ways in which these different types of psychological distress are related to cortisol regulation is not clear. We examined the distinct association of each type of distress with women’s average cortisol level, cortisol awakening response (CAR), cortisol decline across the day (diurnal slope), and overall amount of cortisol secretion across the day (AUC_G_).

**Methods:**

At 6 months postpartum, a diverse group of 58 women completed measures of depression, anxiety, perceived stress, and life stressors. Each woman provided 4 salivary samples for cortisol assay from waking to bedtime on each of 2 consecutive days. Linear regressions were used to examine associations of stress, anxiety and depression to each of the 4 cortisol measures, controlling for number of stressful life events.

**Results:**

Depressive symptoms were associated with less of a rise in the CAR (β = -.46, p = 0.01), steeper diurnal slope (β = .51, p = 0.006), and higher average cortisol level (β = .42, p = .01). Women who met the clinical cutoff for an anxiety disorder had lower overall cortisol output (β = -.29, p = 0.03). Stress was not related to any cortisol metric.

**Conclusions:**

Findings suggest that stress is less associated with cortisol alterations in the postpartum than are more severe types of psychological distress. Anxiety and depression may have distinct and opposite profiles of cortisol dysregulation. Results indicate that mental health assessment is critical even in the later postpartum so that interventions can be initiated to reduce emotional suffering and the risk of impaired cortisol regulation.

## Introduction

Depression, anxiety, and stress are common psychological problems during the postpartum. The overall prevalence of postpartum depression is approximately 17% among healthy mothers without a prior history of depression (Shorey et al. 2018), while postpartum anxiety ranges from 8.5–15% (Dennis et al. 2017; Goodman et al. 2016). A recent study found that 67.6% of women experienced moderate or high postpartum stress (Mollard et al. 2021).

Pregnancy is a time of dynamic hormonal change, including glucocorticoid alterations of the hypothalamic–pituitary–adrenal (HPA) axis (Iliadis et al. 2015; Nazzari et al. 2020; O'Connor et al. 2014). Some of these changes may persist and have been associated with psychological distress in the postpartum (Dickens & Pawluski 2018).

**Fig. 1:**
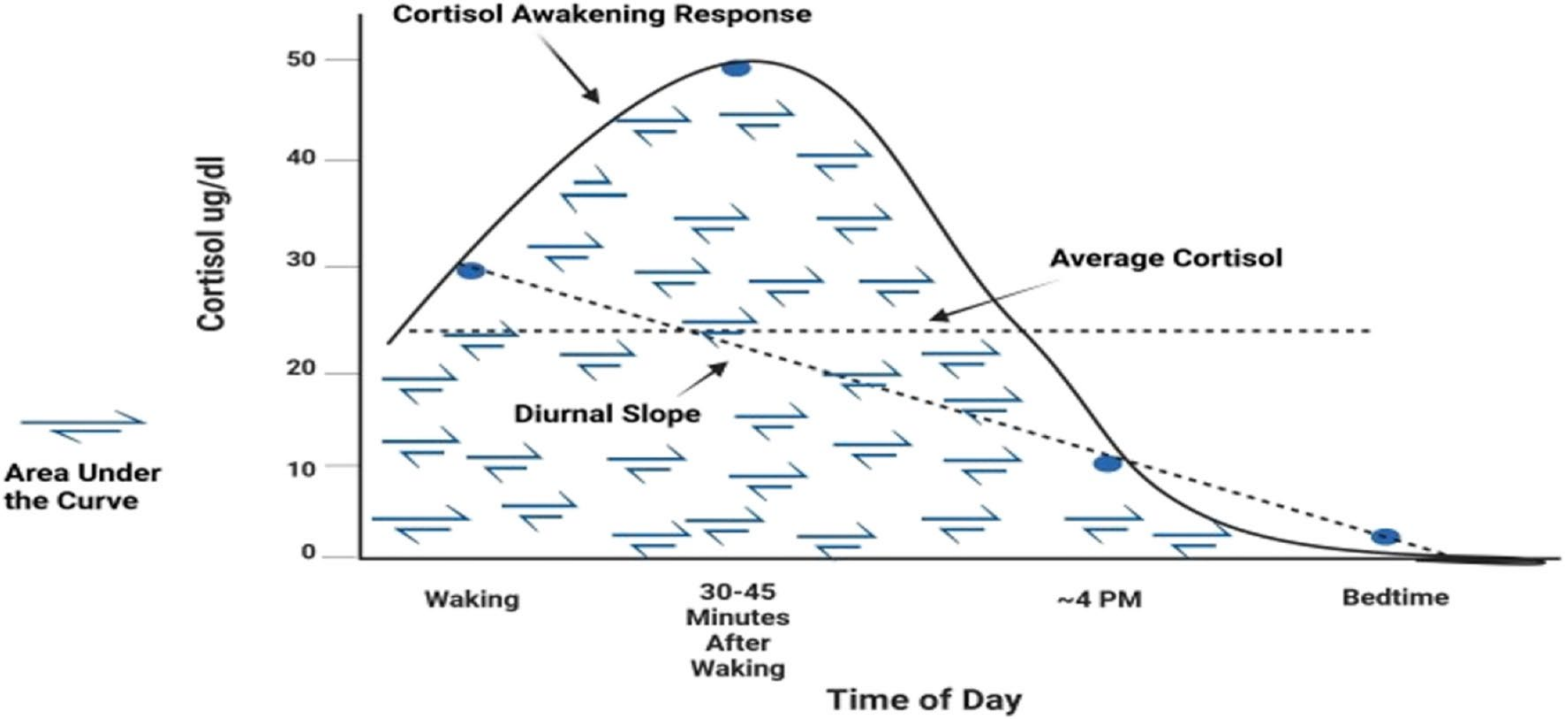
Common Metrics Across the Cortisol Circadian Rhythm

Dysregulation of the HPA axis is hypothesized to be a transdiagnostic mechanism underlying varied types of affective disorders (Belvederi Murri et al. 2016; Hantsoo et al. 2023). Cortisol is the key downstream hormone that is affected by HPA axis dysregulation and can be readily measured. The pattern of cortisol secretion across the day reflects cortisol’s unique circadian rhythm, with key metrics in this rhythm enabling assessment of different irregularities in the cycle (van de Werken et al. 2014). The normative or natural cortisol rhythm is shown in Fig. 1. Cortisol peaks in the early morning, with the highest peak approximately 30–45 min after waking; it then declines throughout the day to its lowest level or nadir around midnight. Four metrics of particular importance to this circadian pattern are highlighted in Fig. 1. They include the *Cortisol Awakening Response* (CAR; the expected rise in cortisol level between waking and 30 to 45 min after waking), the *Diurnal Slope* (the expected decline in cortisol level from waking to bedtime), the overall amount of cortisol secreted throughout the day (*Area Under the Curve*; AUC_G_), and the mean cortisol secretion across the day (*Average Cortisol Level*).

Varied studies have examined relationships of depression, anxiety, and stress to these cortisol markers after the first week postpartum when major hormonal changes begin to stabilize. However, these studies show mixed results.

## Depression and Cortisol

Four studies reported that higher cortisol levels were related to depression during weeks 2 to 8 weeks postpartum (Corwin et al. 2015; Iliadis et al. 2015; Lommatzsch et al. 2006; Pedersen et al. 1993). Conversely, one study found a lower total concentration of morning cortisol was associated with depression (Groer & Morgan 2007). Research during this time has also shown less of a rise in the CAR for women with depression (Taylor et al. 2009).

A few studies assessed the relationship between cortisol and depression from 3 to 12 months after delivery. At three months postpartum, Scheyer and Urizar (2016) found that a flatter slope (less of a decrease in cortisol across the day) was associated with depression. At six months postpartum, two studies reported that higher cortisol levels were associated with depression (Ahn & Corwin 2015; de Rezende et al. 2016), while de Rezende et al. (2016) also observed that depression was related to less of a rise in the CAR. Finally, at 12 months postpartum, Parry et al. (2003) found that lower cortisol was linked to depression. In contrast to these significant associations between cortisol and depression across the first year postpartum, null results were identified by a number of investigators (e.g., Cheng & Pickler 2010; Davis et al. 2007; Field & Diego 2008; Nazzari et al. 2020; Tsubouchi et al. 2011).

## Anxiety and Cortisol

One study collecting data from 2 to 6 weeks postpartum found that anxiety was associated with a higher morning cortisol level (Aparicio et al. 2020), while Galbally et al. (2019) also reported a relationship between anxiety and a higher cortisol level at 12 months postpartum. However, the majority of studies, at various times in the postpartum, observed no association between anxiety and cortisol level (Broeks et al. 2023; Castral et al. 2015; de Rezende et al. 2016; Ghosn et al. 2019; Meinlschmidt et al. 2010; Nazzari et al. 2020).

## Stress and Cortisol

Three studies reported that higher levels of stress were associated with higher cortisol levels, including total cortisol across the day at 6 weeks postpartum (Aparicio et al. 2020), and in the evening at both 3 months (Urizar et al. 2019) and 6 months postpartum (Ahn and Corwin 2015). However, five studies detected no relationship between stress and cortisol level in the postpartum (Bryson et al. 2021; Caparros-Gonzalez et al. 2019; Lang et al. 2021; Scheyer & Urizar 2016; Wang et al. 2022).

## Purpose of the Study

As indicated in the summary above, only a few studies have examined cortisol metrics other than average cortisol level in relation to any type of psychological distress. In addition, from studies to date, it is unclear whether depression, anxiety, and stress (as distinct types of psychological distress) have unique associations with various cortisol metrics. We sought to understand these associations at six months postpartum, a time by which most significant perinatal hormonal changes are thought to have reset to pre-pregnancy levels (Duthie & Reynolds 2013; Ozerdogan et al. 2022).

The purpose of our study was to determine whether depression, anxiety, and stress of women at six months postpartum are related to distinct cortisol metrics (average cortisol level, the CAR, the diurnal slope, or AUC) that may help to explain the unique biological mechanisms associated with these different types of affective distress. By identifying specific alterations that may be markers of different types of distress, we can better assess risk for particular affective disorders. Ultimately, interventions may be developed to normalize alterations associated with a woman’s unique affective problems through precision-based biological or psychological treatments rather than treating all affective problems with a generic, imprecise course of action that may be less optimal for certain types of psychological distress.

## Methods

### Design and Participants

This cross-sectional analysis was part of a larger NIH-funded longitudinal study to assess the effects of varied biopsychosocial risk factors during pregnancy on birth outcomes and stress regulation of infants during early life. Two hundred fifty women were recruited from two obstetric clinics affiliated with a major University during the third trimester of pregnancy and followed until 1 year postpartum. A research coordinator identified women who met criteria for participation and approached them during a scheduled clinic visit to describe the study. Women met inclusion criteria if they were 18 years or older, spoke English or Spanish, and were at potential risk of early delivery (based on obstetrician evaluation). Those with cognitive impairment, adrenal or endocrine disorder, or using a prescribed steroid medication were excluded. Women were given detailed information about the study and informed consent was acquired if they expressed interest in participation.

Saliva samples were collected for cortisol assay from 58 women within this larger cohort; they are the participants whose data are used in this analysis. At 6 months postpartum, they provided four saliva samples each day on two consecutive days and provided questionnaire data for the Perceived Stress Scale, the Patient Health Questionnaire – 9, the Generalized Anxiety Disorders Scale – 7, and the Crisis in Family Systems Interview to measure stressors in their lives.

### Measures

#### Independent Variables

##### The Perceived Stress Scale (PSS)

The PSS (Cohen et al. 1983) measured the degree to which women felt their lives were unpredictable, uncontrollable, and overloaded with stressors over the prior four weeks. Higher scores on the 10-item measure indicate greater perceived stress. Although the PSS does not have validated diagnostic cutoffs, scores from 0–13 have been described as low stress, 14–26 as moderate stress, and 27–40 as high stress (Philpott et al. 2022). The PSS has demonstrated validity and reliability in postpartum women (e.g., Gila-Díaz et al. 2021; Lewis et al. 2021; Murphey et al. 2017).

##### The Patient Health Questionnaire (PHQ-9)

The PHQ-9 was used to assess depression (Kroenke et al. 2002, 2010). Women rated how frequently they had experienced depressive symptoms over the past two weeks. The measure provides total scores for depressive symptoms as well as established clinical ranges, including minimal symptoms of depression (1–4), mild (5–9), moderate (10–14), moderately severe (15–19), and severe (20 +). A score of ≥ 10 has been demonstrated to have both a sensitivity and specificity of 88% for a diagnosis of major depression (Kroenke et al. 2001). In addition to its robust reliability and validity across varied populations, a recent systematic review has shown its psychometric strength in postpartum populations (Wang et al. 2021).

##### Generalized Anxiety Disorder Scale (GAD-7)

We used the GAD-7 to measure women’s degree of anxiety (Spitzer et al. 2006). Like the PHQ-9, it provides a total score as well as clinical cut-points: minimal anxiety (1–4), mild anxiety (5–9), moderate anxiety (10–14), severe anxiety (15 +). A score ≥ 10 has been supported as reflecting a potential diagnosis of anxiety disorder. The measure has shown robust validity and reliability when used in postpartum populations (Howard et al. 2023; Simpson et al. 2014).

### Dependent Variables

#### Salivary Cortisol

A member of the research team reviewed the procedure for collecting saliva with each woman, including actual and pictorial demonstration of the passive drool method for providing saliva and the specific times for sample collection. Women also received written instructions and a phone reminder not to consume food, have alcohol or caffeine, exercise, or take any medications or drugs for 1 h before their saliva sampling. They were asked to rinse their mouths with water 10 min before they provided the sample. Using a cryovial for collection, women gave 1 ml of saliva 4 times a day over 2 consecutive days: upon waking, 45 min after waking, around 4 pm, and prior to bedtime. Salivary samples were stored at -20 °C until shipment to Salimetrics (Carlsbad, CA) for cortisol assay.

Samples were assayed in duplicate using high sensitivity salivary cortisol enzyme immunoassay (ELISA) and run in multiple batches. They were brought to room temperature, vortexed, and centrifuged for 15 min at 3,000 RPM prior to assay. The assay lower limit of sensitivity was 0.007 µg/dl, the standard curve ranged from 0.012–3.0 µg/dl, the intra-assay coefficient of variation was 4.6% and the average inter-assay coefficient of variation was 6%.

Four measures were calculated from the cortisol assays: average cortisol level across the day, Cortisol Awakening Response (CAR), the Diurnal Cortisol Slope (slope), and Area Under the Curve with respect to ground (AUC_G_). As shown in Fig. 1, average cortisol level was the mean cortisol concentration across all samples. The CAR represented the difference between cortisol level from wake time to ~ 45 min following wake time. The slope was the degree of change in cortisol levels across the day from initial waking to bedtime, excluding the second sampling. AUC_G_ was the overall amount of cortisol secreted across the day; we used Pruessner’s trapezoidal formula to calculate AUC with respect to ground (Pruessner et al. 2003).

Values used in analysis for each of the cortisol measures represented the average for that particular metric across the 2 days of cortisol sampling. Averaging cortisol metrics across adjacent days is an established method to enhance reliability of values used in analysis (Adam & Kumari 2009; Adam et al. 2017). Intraclass correlation coefficients (ICC) for the 4 sampling times across the 2 days in this study were: awakening (ICC = 0.74), 30–45 min after waking (ICC = 0.82), 4 pm (ICC = 0.80), and bedtime (ICC = 0.71). These coefficients represented expected levels of reliability considering the recognized day-to-day fluctuations that naturally occur in cortisol activity (Segerstrom et al. 2014).

### Covariates

Because of their previously identified relationships to cortisol, four covariates were examined for potential inclusion in our model testing: adverse childhood events (Epstein et al. 2021; Vacaru et al. 2023), stressful life events over the last 6 months (Karlamangla et al. 2013; Wan et al. 2017), maternal age (García-Blanco et al. 2017; Lindberg et al. 2021), and breast feeding (Cox et al. 2015; Gust et al. 2020). Adverse childhood events were measured with the *Adverse Childhood Experiences Questionnaire* (Felitti et al. 1998) and stressful life events were measured with the *Crisis in Family Systems Interview—Revised* (Berry et al. 2001). Data on maternal age and breast feeding versus formula feeding were based on maternal self-report at the time of data collection.

## Data Analysis

Assumptions for linear regression procedures were assured prior to analyses. Normality was checked with predictive probability plots and the Shapiro-Wilks test to determine if residuals were normally distributed. Linearity was assessed with scatterplots. Multicollinearity of the predictors was also examined by assessing Variance Inflation Factors (VIF). VIF values for stress, depression and anxiety ranged from 1.098 to 1.176, indicating the absence of collinearity. The lack of collinearity among these predictors can also be seen in the moderate correlations between these variables (see Table 2). Cortisol metrics were log transformed to address skew.

To determine the need to adjust for potential covariates in our regression models, we computed preliminary Spearman correlation coefficients between each of the 3 continuous covariates and each of the 4 cortisol metrics. We performed ANOVAs comparing women who breast fed versus formula fed their infant on each of the cortisol metrics. Because of our modest sample size, we included only salient covariates in our regressions that achieved or approached significance in preliminary tests to prevent overfitting our regression models and reducing their statistical power. Only covariates showing an association of p ≤ 0.10 with any cortisol metric were included in all regression models to adjust for their variance.

Separate multiple linear regressions were computed to examine aims for each of the 4 cortisol measures (Average cortisol, CAR, Slope, and AUC_G_). All predictors (perceived stress, depression, and anxiety) as well as any covariate that met the p ≤ 0.10 criterion (e.g., stressful life events) were included together in each of the cortisol models we tested.

In our first set of analyses, we regressed the cortisol measure being examined on continuous scores for the stress, depression, and anxiety predictors. In a second set of analyses, we regressed cortisol on categorical, clinical cut-offs for the predictors whereby women who met criteria for diagnostic referral (scores of ≥ 10 for depression and anxiety), or for moderate or greater stress (scores of 14 or more), were compared to those who did not meet these criteria.

## Results

Characteristics of the sample are shown in Table 1. 55% (n = 32) of the women were diverse in heritage (i.e., having African, Asian or Hispanic/Latina descent). Most were in committed relationships (86%) and employed full time (63%). Although 60% were college educated, 42% of the women relied on some form of government assistance, such as housing, food, or childcare support. On average, women reported mild depression (M = 4.53), mild anxiety (M = 5.80), and moderate stress (M = 14.19). However, 15% met the clinical cutoff for depression on the PHQ-9 and 20% for anxiety on the GAD-7.

**Table 1:** Characteristics of Women Participating in the Study [N = 58]

	Mean [SD]	Median	n	%
Age in Years	34 [5.5]	35		
Race/Ethnicity				
African American/Black			11	19%
Asian American			11	19%
Euro-American/White			36	63%
Latina/Hispanic			10	17%
Relationship Status				
Committed Partner			50	86%
Single			7	13%
Separated/Divorced			1	1%
Education				
High School or Less			12	20%
Some College			12	20%
College Graduate			13	23%
Advanced Degree			21	37%
Employment				
Unemployed			13	23%
Part time			8	14%
Fulltime			37	63%
Reliance on Government Assistance				
Yes			24	42%
No			34	58%
Stressful Events (Number)	4.36 [3.85]	5.00		
Perceived Stress	14.19 [6.52]	14.50		
Depression	4.53 [4.67]	4.22		
Anxiety	5.80 [4.98]	5.28		
Clinically Significant Stress			28	49%
Clinically Significant Depression			9	15%
Clinically Significant Anxiety			12	20%
Average Cortisol Level	.331 [.83]	.364		
Cortisol Awakening Response	-.040 [.17]	-.038		
Cortisol Diurnal Slope	.350 [.10]	.303		
Cortisol Area Under the Curve	250.15 [79.40]	202.12		

SD = standard deviation; n = number of women

In preliminary analyses of the associations between potential covariates and cortisol metrics, only stressful life events showed relationships to cortisol that met the p =  ≤ 0.10 criterion for inclusion in the regression analyses. Stressful events were correlated with higher average cortisol (r = 0.20, *p* = 0.05) and a steeper slope (r = 0.19, *p* = 0.06). Childhood adverse experiences had correlation coefficients ranging from r = 0.02 (*p* = 0.85) for average cortisol to r = -0.14 (*p* = 0.18) for the CAR. Correlations for age ranged from r = 0.02 (*p* = 0.84) for AUC to r = . 14 (*p* = 0.15) for diurnal slope. Differences in cortisol measures between women who breast fed (n = 32) and formula fed (n = 26) their infants ranged from F = 0.66 (*p* = 0.74) for the CAR to F = 0.11 (*p* = 0.48) for average cortisol. Based on these associations, only the variable of stressful life events was controlled for in regression analyses.

Bivariate correlations for all key study variables are shown in Table 2. A greater number of stressful life events was associated with more symptoms of anxiety and depression as well as higher average cortisol. The 3 measures of psychological distress all had moderate relationships with one another (ranging from r_s_ = 0.45 to r_s_ = 0.64). Stress had no significant associations with any cortisol metric while anxiety was positively related to greater average cortisol (r_s_ = 0.20, p = 0.05) and depression was associated with a decreased CAR (r_s_ = -0.23, p = 0.05).

**Table 2:** Spearman Correlations between Study Variables

	Psychological Distress			Cortisol Metrics			
	Stress	Anxiety	Depression	Average	CAR	Slope	AUC_G_
Stressful Events	.17	.25**	.20**	.20**	-.04	.19*	.13
Stress (Perceived)		.55***	.64***	.07	-.15	-.16	.04
Anxiety			.45***	.20**	-.17	-.11	.06
Depression				.09	-.23**	-.06	.05
Average Cortisol					.14	.41***	.87***
CAR						-.13	-.13
Diurnal Slope							.18*

CAR = Cortisol Awakening Response; AUC = Area Under the Curve with Respect to Ground

^*^p = .10; **p = .05; ***p = .01

Linear regressions, using continuous scores for depression, anxiety, and stress, produced significant results only for depression and cortisol. As shown in Table 3, neither stress nor anxiety were associated with any of the cortisol measures. However, more depressive symptoms were significantly associated with a higher average cortisol level (β = 0.42, p = 0.01), greater cortisol AUC (β = 0.35, p = 0.04), and a steeper diurnal slope (β = 0.51, p = 0.006). More severe depressive symptoms were also associated with a decreased CAR (β = -0.46, p = 0.01). When using diagnostic/clinical cutoffs as the predictor (i.e., whether women met the cut point for diagnostic referral or not), no type of psychological distress (i.e., depression, anxiety or stress) was associated with the CAR (Table 4). However, women who met the cutoff for a potential diagnosis of depression (PHQ-9 ≥ 10) had a higher average cortisol level (β = 0.31, p = 0.02) and a steeper diurnal slope (β = 0.40, p = 0.003) than women who did not meet the cutoff. In direct contrast, women who met the cutoff for a potential diagnosis of anxiety (GAD-7 ≥ 10) had a lower average cortisol level (β = -0.30, p = 0.03) and a lower AUC (β = -0.29, p = 0.03) than women who did not meet the cutoff.

**Table 3:** Linear Regression Models for the Relationships of Stress, Depression & Anxiety Symptoms to Cortisol Metrics*

				Beta	SE	p-value	95% Confidence Interval
Average Cortisol Level							
Stressors				.10	.00	.45	-.006, .012
Stress				-.12	.01	.50	-.026, .013
Depression				.42	.01	.01	.007, .061
Anxiety				-.23	.01	.14	-.039, .006
Cortisol Awakening Response							
Stressors				.12	.07	.38	-.085, .218
Stress				.09	.01	.64	-.011, .017
Depression				*-*.46	.01	.01	-.045, -.006
Anxiety				-.10	.01	.51	-.020, .010
Diurnal Slope							
Stressors				-.21	.00	.12	-.022, .003
Stress				-.22	.01	.22	-.041, .009
Depression				.51	.02	.006	.019, .090
Anxiety				-.19	.01	.23	-.049, .012
Area Under the Curve							
Stressors				.16	.00	.12	-.004, .016
Stress				-.07	.01	.69	-.025, .017
Depression				.35	.02	.04	.001, .060
Anxiety				-.28	.01	.08	-.046, .003

^*^Adjusting for Life Stressors

**Table 4:** Linear Regression Models for the Relationships of Diagnostic/Clinical Cutoffs to Cortisol Metrics*

	Beta	SE	p-value	95% Confidence Interval
Average Cortisol Level				
Stressors	.11	.00	.39	-.005, .013
Stress Cutoff	-.02	.09	.83	-.203, .164
Depression Cutoff	.31	.15	.02	.067, .675
Anxiety Cutoff	-.30	.12	.03	-.510, -.028
Cortisol Awakening Response				
Stressors	.02	.00	.89	-.006, .007
Stress Cutoff	-.14	.06	.28	-.205, .060
Depression Cutoff	-.12	.11	.37	-.320, .121
Anxiety Cutoff	.03	.08	.86	-.160, .190
Diurnal Slope				
Stressors	-.25	.01	.06	-.023, .001
Stress Cutoff	-.07	.12	.58	-.306, .174
Depression Cutoff	.40	.21	.003	.228, 1.070
Anxiety Cutoff	-.24	.15	.08	-.591, .039
Area Under the Curve				
Stressors	.17	.01	.22	-.004, .016
Stress Cutoff	.01	.10	.93	-.192, .208
Depression Cutoff	.24	.16	.07	-.027, .637
Anxiety Cutoff	-.29	.13	.03	-.547, -.020

^*^Adjusting for Life Stressors

## Discussion

Findings from our study suggest that different types of psychological distress in the postpartum are linked to women’s cortisol in distinct ways. Having more depressive symptoms was associated with less of a rise in the CAR, a greater decline in cortisol across the day (steeper diurnal slope), greater overall amount of cortisol secretion across the day, and higher average cortisol level. In contrast, meeting the criteria for a potential anxiety disorder was associated with lower average cortisol level and less overall amount of cortisol secretion across the day. Lastly, symptoms of stress were not related to any cortisol metric.

### Depression and Cortisol

Our finding that a blunted CAR (i.e., less of a rise or surge in cortisol after waking) was associated with depression is consistent with results of de Rezende et al. (2016) at six months postpartum and Taylor et al. (2009) at 7.5 weeks postpartum. Although their study occurred at 2 weeks postpartum, Corwin et al. (2015) found, as did we, that depression is related to a higher average cortisol level.

In contrast to these areas of congruence with other studies, our results indicating that a steeper (greater) cortisol decline across the day is linked to greater depression differ from previous research. Scheyer and Urizar (2016) reported depression being associated with a flatter diurnal slope (i.e., less decline in cortisol across the day). In our findings, a steeper slope was associated with more depressive symptoms and it distinguished women who met the cutoff for clinically significant depression from those who did not. The difference in our findings from those of Scheyer and Urizar could be related to characteristics of our sample populations, cortisol collection methods, depression measures that were used, not adjusting for stress and anxiety, or their data collection at 3 months postpartum rather than six months as we did.

### Anxiety and Cortisol

Our results suggest that anxiety and depression have opposite profiles of cortisol dysregulation. In contrast to the higher levels of cortisol for depressed women, women who met the cutoff for a clinically significant anxiety disorder had a lower average cortisol level than women who did not meet the cutoff. Our results differ from Aparicio et al. (2020) who reported a positive association between anxiety and cortisol levels. However, that study assayed cortisol from breast milk collected at one timepoint in the morning at 6 weeks postpartum; and they measured continuous anxiety scores (not cutoffs for a potential anxiety diagnosis).

One notable aspect of our results when comparing anxiety and depression findings is that anxiety was only associated with cortisol alterations when we compared women meeting a cutoff for an anxiety disorder with those who did not. This may suggest that lower average cortisol and less overall cortisol secretion reflect more severe anxiety symptoms and do not necessarily emerge with moderate or minimal anxiety. It is possible that the anxiety of women who met the cutoff had persisted for an extended period of time until becoming more severe. In contrast to anxiety, cortisol alterations associated with depression were consistently observed: both as depressive symptoms increased from mild to severe, and when comparing more severely depressed women who met the diagnostic cutoff with those who did not.

Effects of comorbid anxiety and depression need also to be studied since these mental health problems share a high level of genetic risk and frequently co-occur (Kalin 2020). The cortisol profile of women who have more severe postpartum symptoms of both depression and anxiety may be uniquely different from those seen in women who have only depression or anxiety. It has been reported in previous research that comorbid anxiety/depression was associated with elevated cortisol during pregnancy, although depression or anxiety alone showed no relationships to cortisol (Evans et al. 2008). Unfortunately, we could not examine the association between postpartum comorbidity and cortisol in our study since only 2 women in the sample met clinical cutoffs for both depression and anxiety.

### Stress and Cortisol

Our findings suggest that stress is less associated with cortisol alterations at 6 months postpartum than are more severe types of psychological distress such as depression and anxiety. The lack of association we observed between postpartum stress and any cortisol metric corroborates results of previous studies (Bryson et al. 2021; Caparros-Gonzalez et al. 2019; Lang et al. 2021; Scheyer & Urizar 2016). Although it is often assumed that perceived stress and cortisol are integrally linked, evidence continues to indicate that they measure distinct components of the stress response and are not necessarily always related (Leung & Kyung 2022; Musana et al. 2020), including in the postpartum (Urizar et al. 2019).

## Limitations

Our study's smaller sample size limits the ability to generalize to larger populations of postpartum women. Although we provided detailed instructions for the times when salivary samples were to be collected, we did not use MEMS caps or other technology to automatically record actual times when samples were provided. Adjusting for times of awakening and going to bed could account for some of the variance in the regression models. Because of the cross-sectional nature of the research, it is not possible to know the trajectory of potential change in the relationship between psychological symptoms and cortisol regulation from prior to pregnancy through the first postpartum year. Lastly, we were not able to include clinician-based diagnoses as an adjunct to our symptom assessments.

## Conclusions

Our cortisol findings for postpartum depression and anxiety suggest dysregulation of the Hypothalamic–Pituitary–Adrenal (HPA) axis for women experiencing more severe symptoms at 6 months. The association between depression and higher cortisol levels could reflect over-secretion of hormones at various points in the HPA axis or impaired glucocorticoid negative feedback (Hantsoo et al. 2023; Pariante & Lightman 2008). In addition, the diminished rise in CAR but steeper decline in cortisol across the day for women with greater depression could indicate a potential impairment in the circadian cortisol system (Clow et al. 2009; Dedovic & Ngiam 2015). The surge of cortisol reflected in the CAR is thought to prepare the body physiologically to deal with upcoming demands of the day (Smith et al. 2020). Thus, more depressed women whose rise in CAR is diminished may have difficulty mounting this preparatory state. A blunted, diminished rise in CAR has shown consistent links to hopelessness, persistent sadness and other negative emotional states (Dedovic & Ngiam 2015). However, the implications of a steeper cortisol slope are not clear since it has been associated with both high arousal positive affect (Hoyt et al. 2015) and less optimal emotional states such as negative affect and loneliness (Drake et al. 2016; Giese-Davis et al. 2006). Further research is needed to understand the ways in which these cortisol alterations may interact synergistically with one another and either result from or contribute to postpartum depression.

The lower average levels and reduced amount of cortisol secretion across the day found for women with more severe anxiety suggest a down-regulation of their HPA axis. The negative feedback mechanism of the HPA axis may become overactive when anxiety is more severe, leading to attenuated or reduced HPA axis activity as a somewhat normative state. Such HPA axis down-regulation has been attributed to potential sensitization of receptor responses or adrenal exhaustion, among other potential processes (Adam et al. 2017; Herman et al. 2016). Future research needs to examine the role of anxiety prior to and during pregnancy in contributing to anxiety-related cortisol regulation in the postpartum.

Our findings also highlight the importance of not confounding distinct psychological states of stress, anxiety and depression; these mood states demonstrated unique associations with cortisol metrics that have differential implications for targeted and appropriate intervention. For instance, the classes of medication required to stimulate cortisol production when addressing its down-regulation are uniquely different from those required to inhibit its secretion when cortisol is too high. Likewise, understanding the nuanced character of a cortisol impairment could: a) inform the therapeutic goals of biofeedback or a chronobiologic intervention, or b) guide the choice between behavioral activation and stress reduction to assure the best match for a woman’s unique hormonal needs. Thus, by identifying precise cortisol alterations that are associated with discrete types of affective distress (e.g., anxiety versus depression), we can better assess risk for particular affective disorders and develop interventions to normalize HPA axis impairments that are targeted to the exact nature of the cortisol dysregulation. Although such applications motivate our work, substantial research is still needed before they can be realized.

Lastly, in many settings, mental health assessment ceases or is not considered after 3 months postpartum. Our findings suggest that assessment for affective symptoms is still critical at 6 months postpartum, with 15–20% of women in our sample meeting the cutoffs for anxiety or depression. Interventions can then be initiated to reduce emotional suffering and the risk of impaired cortisol regulation.

## Data Availability

The data that support the findings of this study are available from the corresponding author [SJW] upon reasonable request.
